# Febrile Temperature Elevates the Expression of Phosphatidylserine on *Plasmodium falciparum* (FCR3CSA) Infected Red Blood Cell Surface Leading to Increased Cytoadhesion

**DOI:** 10.1038/s41598-018-33358-2

**Published:** 2018-10-09

**Authors:** Rou Zhang, Rajesh Chandramohanadas, Chwee Teck Lim, Ming Dao

**Affiliations:** 1 Singapore-MIT Alliance, National University of Singapore, Singapore, 117576 Singapore; 2 Infectious Disease IRG, Singapore-MIT Alliance for Research and Technology (SMART) Centre, Singapore, 117543 Singapore; 3 Department of Biomedical Engineering, National University of Singapore, Singapore, 117576 Singapore; 4 Engineering Product Development Pillar, Singapore University of Technology & Design, 20 Dover Drive, Singapore, 138682 Singapore; 5 Mechanobiology Institute, National University of Singapore, 5A Engineering Drive 1, Singapore, 117411 Singapore; 6 Biomedical Institute for Global Health Research and Technology, National University of Singapore, MD6, 14 Medical Drive, #14-01, Singapore, 117599 Singapore; 7 Department of Materials Science and Engineering, Massachusetts Institute of Technology, Cambridge, MA 02139 USA

## Abstract

During the asexual intra-erythrocytic cycle, *Plasmodium (P.) falciparum* exports parasitic proteins to the surface of infected red blood cells (iRBCs) facilitating its cytoadhesion to various endothelial host receptors. This adhesive behavior is a critical contributor towards disease manifestation. However, little is known about the influence of recurring elevated temperature – a common symptom of the malaria infection – on the adhesive properties of iRBCs to endothelial receptors. To address this, we performed dual-micropipette step-pressure technique between *P. falciparum* (strain FCR3CSA) iRBCs and Chinese Hamster Ovary cells expressing Chondroitin sulfate A (CHO-CSA) after transient iRBCs incubation at febrile temperatures which revealed increase in adhesion parameters. Furthermore, flow cytometry analysis revealed an increase in phosphatidylserine (PS) expression on the iRBC surface following exposure to febrile temperature. The adhesion between iRBCs and CHO-CSA cells was remarkably reduced in presence of soluble Annexin V, indicating the mediation of PS on the adhesion events. Our results suggest that elevated PS recruitment on iRBC under thermally stressed conditions contributes to the increased adhesive behavior of iRBCs CSA-binding phenotype to CHO-CSA.

## Introduction

Malaria is the most prevalent blood-borne infectious disease caused by protozoan parasites of the species *Plasmodium*. About half of the world’s population inhabits a malaria endemic regions. In 2016, 216 million malaria infections were reported resulting in 445,000 deaths^1^. During the intra-erythrocytic asexual cycle, *Plasmodium falciparum*, the most common causative agent of malaria-associated pathology in humans, modifies the infected red blood cells (iRBCs) extensively. IRBCs become less deformable and eventually adhere to the blood vessel walls to avoid clearance by the spleen^2–6^. The adherence of iRBCs to blood vessel walls induces microvasculature obstruction, reduces microcirculatory flow and can cause a fatal outcome.

To establish adherence to the endothelial cells, *P. falciparum* rely on several parasite proteins that are exported to the surface of iRBCs. The *P. falciparum* erythrocyte membrane protein 1 (*Pf*EMP1) is one such adhesive ligand exported to the iRBC membrane. It is encoded by the ‘*var’* gene family and contains multiple unique domains to facilitate efficient binding to a variety of host receptors including CD36, ICAM1 and CSA^7,8^. Several other adhesive ligands, such as RIFIN, STEVOR and ring surface protein 2 (RSP-2) have also been identified as key molecules contributing to the increased adhesive behavior of iRBCs^9–13^. Moreover, previous studies reported that a phospholipid component residing on the inner-leaflet of the red blood cell (RBC) lipid bilayer, phosphatidylserine (PS), to significantly enhance the RBC adhesiveness (in both healthy and infected RBCs) particularly to TSP and CD36^9,14,15^. Phosphatidylserine can be flipped out to the surface of RBCs upon long-term exposure (>24 h) to thermally or oxidatively stressed condition^15–17^.

The binding strength and kinetic parameters between host receptors and corresponding ligands provide important information towards better understanding of iRBC sequestration. Several studies have quantified the binding strength between *Pf*EMP1 and different host receptors^18–20^. These reports predominantly outline the interactions at room temperature (23 °C) and at normal body temperature (37 °C), whereas the effect of fever is less explored within the context of receptor-ligand interactions. Importantly, the elevated temperatures during fever is likely to alter multiple molecular pathways required for the survival of malaria parasites^21^.

Here we study the effect of a transient exposure to febrile temperatures on the cell-cell adhesion strength via a dual-pipette step-pressure technique. We first measured the binding strength and the percentage of binding between *Pf*EMP1 and CSA bearing Chinese Hamster Ovary (CHO-CSA) cells and compared our observations with previously published results regarding the interactions between *Pf*EMP1 and other host receptors. Further, we evaluated the effect of febrile temperature on iRBCs adhesion, which revealed significant increase in iRBC adhesion to target cells. Moreover, a transient exposure to febrile temperatures increased the expression of phosphatidylsesrine (PS) on iRBC surface. Using soluble Annexin V, we specifically blocked PS expression on iRBC surface, which subsequently resulted in a remarkable reduction in the iRBCs’ adhesion forces to CHO-CSA cells.

## Materials and Methods

### Culture and maintenance of adhesive malaria parasites

*Plasmodium falciparum* strain FCR3CSA was used in this study. Parasites were maintained in human RBCs supplemented with human serum (0.5% wt/vol) in HEPES-buffered RPMI media (malaria culture medium), supplemented with hypoxanthine (50μg mL^−1^), NaHCO_3_ (25 mM), gentamicin (2.5μg mL^−1^). Continuous culture methods were followed as described in previous studies^22^. Parasites were synchronized by treatment with 5% D-sorbitol (Sigma) to select ring-stage infections, and adhesive parasites of trophozoite stage were selected periodically using CHO-CSA cells. Fresh malaria culture medium was added with 5% hematocrit for continuous culture.

### Culture of CHO cells

CHO-CSA (CHO-K1:ATCC CCL-61) were cultured in CHO cell culture medium of 90% F-12K Medium (with L-Glutamine, ATCC), 9% Fetal Bovine Serum (Origin: USDA, PAA, de-activated), and 1% pen-streptomycin. Sub-culture was performed every two days at 80% confluence. For sub-culture, cells were incubated in 5 ml Accutase Cell Detachment Solution (Innovative Cell Technologies, Inc.) at 37 °C for 10 min. The detached CHO cells were then washed three times by centrifugation (1500 rpm, 5 min) in RPMI-1640. The supernatant was removed and cells were resuspended in 5 ml culture medium at 2 × 10^4^ cells/ml. CHO-CSA cells were not used beyond 25 passages.

### Febrile temperature incubation and adhesive sample preparation

Trophozoite stage parasites (30–32 hours post-infection) were resuspended in malaria culture medium and incubated in a water bath pre-set at 40 °C for a period of 1 h. Cells were then washed in RPMI-1640 and resuspended in PBS with 500 μg/ml BSA at 5 × 10^5^ cells/ml. Another aliquot of the same parasite culture incubated at 37 °C served as control.

CHO-CSA cells were detached using 5 ml of Accutase Cell Detachment Solution (Innovative Cell technologies, Inc.) for a T25 flask and washed twice in RPMI-1640. CHO-CSA cells were then resuspended at 10^5^ cells/ml in PBS with 500 μg/ml BSA together with iRBCs that were differentially exposed to febrile condition.

### Dual pipette assay and step-pressure technique

The cell-cell adhesion force between the iRBCs and CHO-CSA cells was measured using the dual-pipette assay and step-pressure technique^18,20,23^. To fabricate the micropipettes, borosilicate glass tubings (B100-75-10, Sutter) were pulled using Sutter Micropipette Puller (Sutter Instruments) and forged by Narishige Microforge (MF900, Narishige). Micropipettes with inner diameter (ID) of 5 to 10 μm were used to hold the CHO-CSA cells. Smaller micropipettes of 1 to 2 μm were fabricated to manipulate the iRBCs and to measure the adhesion force.

**Figure 1:**
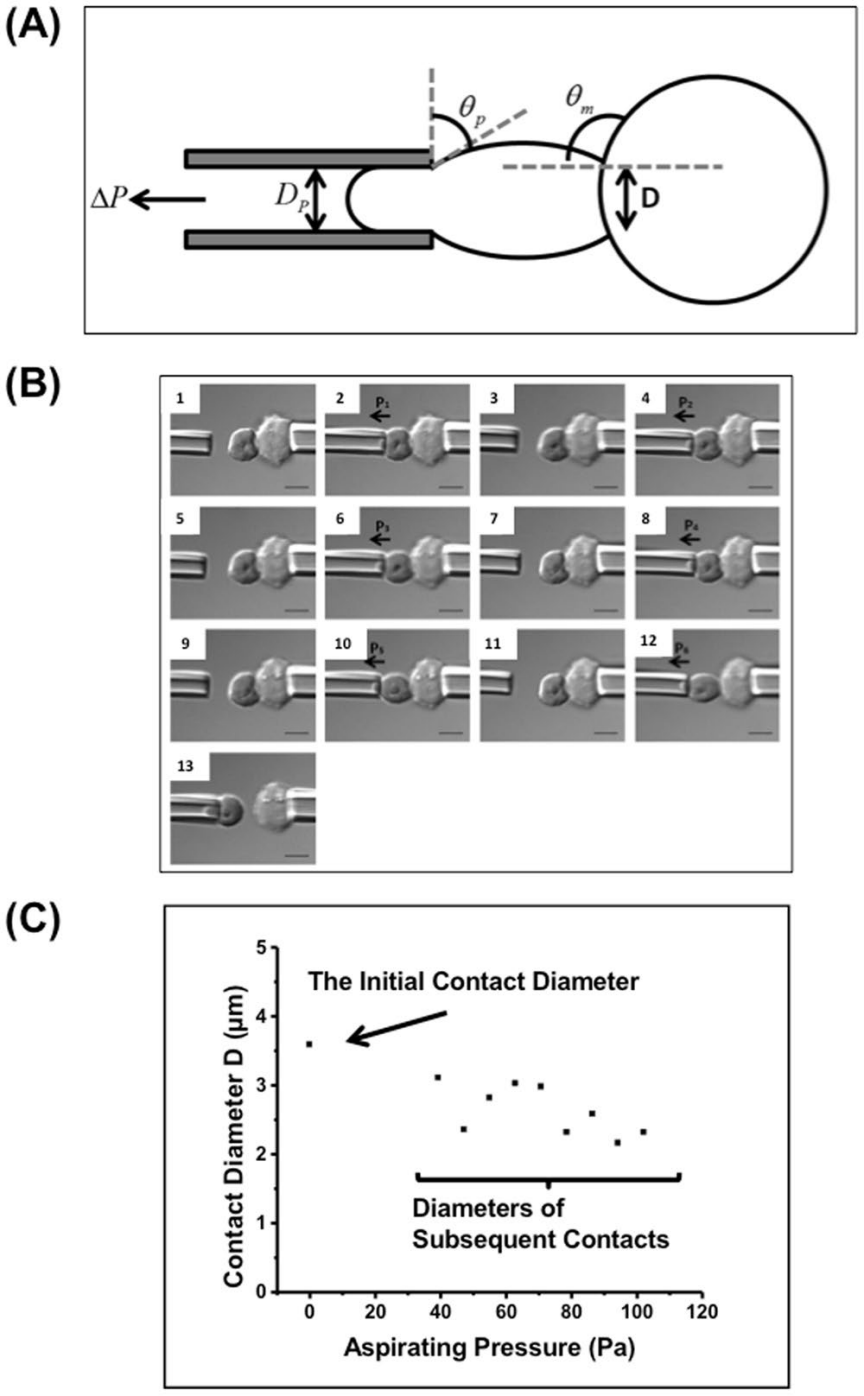
Febrile temperature greatly enhanced cell-cell adhesion, and the contact diameter between cells. (**A**) Schematic illustration of the geometry of one iRBC (left) being separated from the CHO cell (right), and the calculation of adhesion force and energy density. (**B**) DIC images of one iRBC (left) being detached from the adhesive CHO cell (right). The aspirating pressure increased stepwise in each pulling (scale bar = 5 μm) (**C**) The relationship between the contact diameter and the aspirating pressure. Each data point represents the cell-cell contact diameter when the iRBC was pulled at corresponding aspirating pressure. Data was extracted from one experiment.

The mixture of treated iRBCs and CHO-CSA cells was loaded into a cell mounting chamber made of two coverslips and parafilm separate gasket. A CHO-CSA cell was held by a larger micropipette (ID of 5 to 10 μm) as the adhesive target. An iRBC was then held and manipulated by a smaller micropipette (ID of 1 to 2 μm). The aspiration pressure exerted by the micropipettes could be adjusted by the height of the externally connected water column. The iRBCs were kept in touch with the target cells for 30 s to allow a stable cell-cell adhesion. The adherent iRBCs were then removed by the micropipette at increasing pressure (Fig. 1(B)). The suction pressure to separate the two cells was measured by a pressure transducer (P55, Validyne Engineering). Optical images were viewed using a 100× oil objective with DIC and captured by Olympus QColor5 High Resolution Color CCD Digital FireWire Camera. Images were processed by QCapture Pro 6.0.

Force measurements were conducted at 37 ± 0.5 °C. A heating plate (Linkam Scientific Instruments, UK) and an objective heater (Tokai Hit, Japan) were used to maintain the temperature within the required range. A thermocouple thermometer was used to continuously monitor the testing medium temperature.

### Calculation of Adhesion Force

The aspiration pressure ΔP to separate the two cells was recorded, and the adhesion force was calculated as,

1 $${\rm{F}}={\rm{\Delta }}{\rm{P}}{\rm{\pi }}{({{\rm{D}}}_{{\rm{P}}}/{\rm{2}})}^{{\rm{2}}}$$

where F is the adhesion force, ΔP is the negative aspirating pressure provided by the micropipettes, and D_P_ is the inner diameter of the micropipette (Fig. 1(A)). As the lowest negative pressure to be applied was 7.848 pa using the step-pressure technique, the threshold force measurement was calculated as 14 pN if a 1.5 µm micropipette was used.

### Calculation of the adhesion energy density

The adhesion energy density w_a_ is defined as the energy to separate a unit contact area. The Young equation was used to calculate the adhesion energy density of the cell-cell adhesion interface^24–28^,

2 $${{\rm{w}}}_{{\rm{a}}}={{\rm{T}}}_{{\rm{m}}}({\rm{1}}-{\cos {\rm{\theta }}}_{{\rm{m}}})\approx {{\rm{T}}}_{{\rm{m}}}$$

where T_m_ is the membrane tension close to the contact rim, and θ_m_ is the angle between the CHO cell membrane and the horizontal (Fig. 1(A)). The membrane tension T_m_ was calculated as,

3 $${{\rm{T}}}_{{\rm{m}}}={\rm{F}}/({\rm{\pi }}D\,\sin \,{{\rm{\theta }}}_{{\rm{P}}})$$

where F is the adhesion force, D is the cell-cell contact diameter, and θ_P_ is the angle between the iRBC membrane and the vertical (Fig. 1(A)).

In most cases θ_m_ is close to 90°, and the adhesion energy density is close to T_m_.

### PfEMP1 expression

The staining of iRBCs for *Pf*EMP1 expression levels followed the protocol as described previously^29^. After incubating at 40 °C, trophozoite stage parasites were washed in RPMI-1640 and resuspended in PBS with 500 μg/ml BSA at 10^6^ cells/ml. This sample was then incubated with a mouse monoclonal antibody (mAb) against VAR2CSA *Pf*EMP1 cysteine-rich interdomain region for 30 min at 4 °C. After centrifugal separation, the iRBCs were washed and incubated with Alexa Fluor 594-conjugated anti-mouse immunoglobulin G (Invitrogen) for 30 min. SYTO16 (Invitrogen) was used to stain the DNA contents of iRBCs. Parasites were then fixed in 4% freshly prepared paraformaldehyde for flow cytometry analysis. A control group maintained at 37 °C was also included in all experiments. The immunofluorescent intensity (MFI) of Alexa Fluor 488 was measured by flow cytometry.

### PS expression

The staining of phosphatidylserine (PS) on the iRBC membrane followed the protocol described in the previous study^17^. Annexin V:FITC Apoptosis Detection Kit I (BD Pharmingen) was used to monitor the PS expression on the iRBC surface. After incubating at 40 °C, iRBCs were washed in PBS with 500 μg/mL BSA and resuspended in 1× Annexin V Binding Buffer (BD Pharmingen) at 1 × 10^6^ cells/ml. Infected RBCs were incubated at room temperature for 15 min with FITC Annexin V diluted 1/100 together with Dihydroethidium, HE (Invitrogen) diluted 1/100. After washing with PBS that contains 500 μg/ml BSA, PS expression was examined using flow cytometry (wavelength: 488 nm). A group of iRBCs that were maintained at 37 °C served as controls.

### PfEMP1 and PS inhibition assay

Soluble CSA (Sigma-Aldrich) was used to block the adhesion mediated by *Pf*EMP1. After incubating at 40 °C for 1 h, parasites were washed in RPMI-1640 and resuspended in PBS with 100 μg/ml CSA and 400 μg/ml BSA at 5 × 10^5^ cells/ml. A control group was prepared by re-suspending parasites in PBS with 500 μg/ml BSA.

The adhesion mediated by PS was inhibited by Annexin V (BD Pharmingen). After the febrile temperature incubation, iRBCs were washed in RPMI-1640 and resuspended in freshly prepared PS inhibition medium (450 μg/ml BSA and 50 μg/ml Annexin V in PBS with Ca^2+^) at 5 × 10^5^ cells/ml. A control group was prepared by re-suspending iRBCs in PBS with 500 μg/ml BSA.

To block the adhesion mediated by both PS and *Pf*EMP1, iRBCs were washed and re-suspended in PBS with 350 μg/ml BSA, 50 μg/ml Annexin V and 100 μg/ml CSA at 5 × 10^5^ cells/ml. A control group was prepared by resuspending iRBCs in PBS with 500 μg/ml BSA.

### Statistical Analysis

Scatter dot plots with median value of each group were used for data presentation. Statistical significance and non-Gaussian distribution were determined by the Kruskal–Wallis one-way analysis of variance with Dunns post hoc test and Mann-Whitney test respectively.

## Results

### Exposure to Febrile temperature significantly increases iRBC adhesion to Chondroitin Suphate A expressing CHO cells

**Figure 2:**
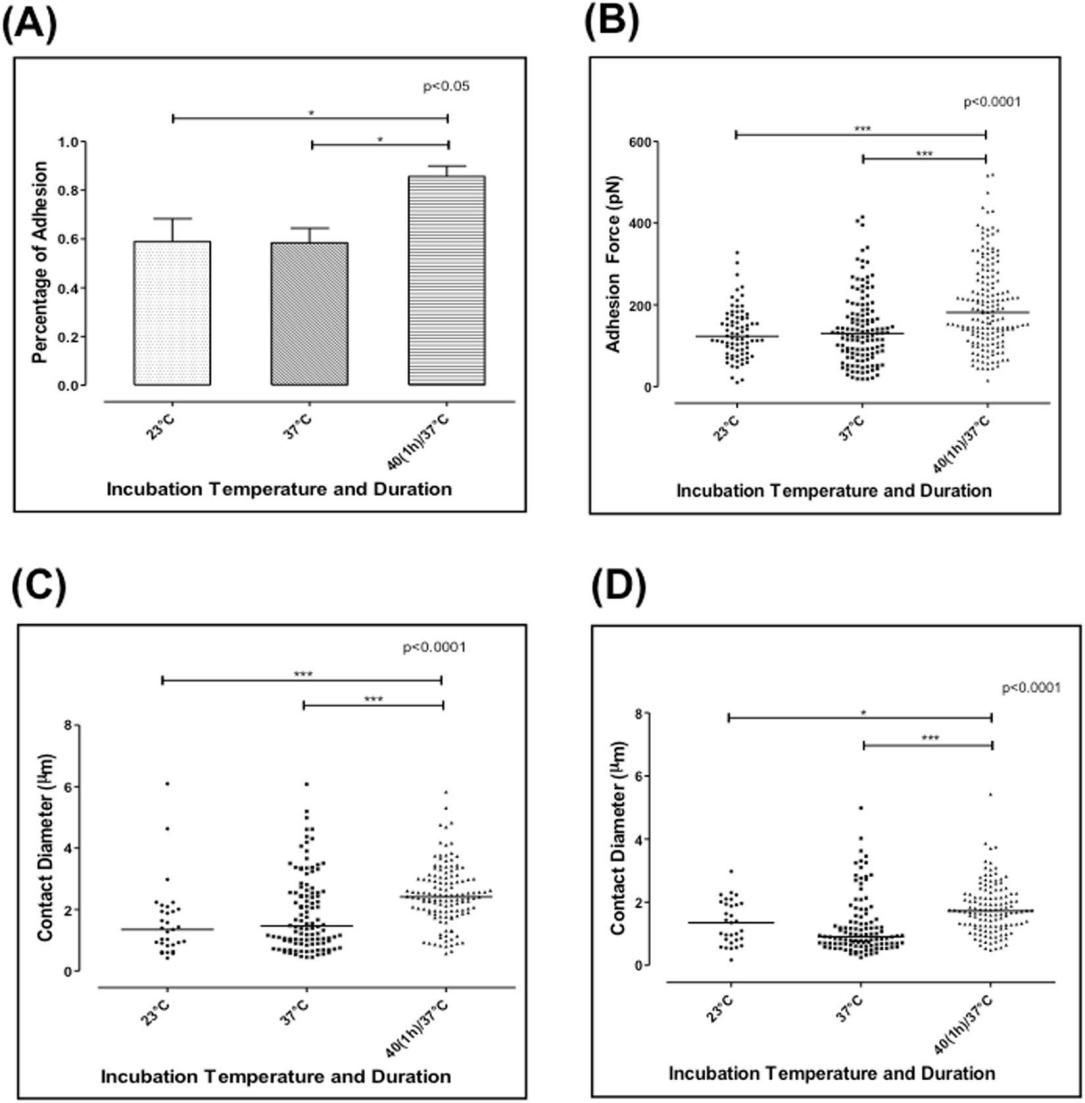
(**A**) Percentage of adhesion measured at 23 °C, 37 °C, and after 1 h or 2 h incubation at 40 °C (n = 5). (**B**) The adhesion force measured at 23 °C, 37 °C, and after 1 h incubation at 40 °C. (**C**) The initial contact diameter measured at 23 °C, 37 °C, and after 1 h incubation at 40 °C. (**D**) The final contact diameter before the two cells were separated, measured at 23 °C, 37 °C and after 1 h incubation at 40 °C. Each point in (**B**), (**C**) and (**D**) represents the adhesion force of one cell pair, and the bar represents the median value of each data set. Krauskal-Wallis Test and Dunn’s post hoc test.

To study the effect of febrile temperature on adhesion, iRBCs were subjected to 1 h incubation at 40 °C before conducting the dual-pipette adhesion experiments. Trophozoite stage parasites are susceptible to febrile temperatures. Longer incubations (of more than 2 h) may lead to parasite death^21^. Thus, 2 h incubation were used to study the fever temperature effect on live iRBCs adhesion. The adhesion measurements were performed at 37 °C after the incubation of iRBCs at 40 °C. Five independent experiments of force measurement were conducted as part of this analysis. In total, 181 iRBCs after 1 h febrile temperature incubation were measured. In addition, 75 iRBCs incubated at room temperature (23 °C), and 223 iRBCs incubated at 37 °C were measured as control groups. All measurements were done within 1 h of treatment to avoid artefactual effects arising from liquid evaporation etc. under the conditions of measurements. Moreover, experiments were repeated to obtain reliable measurements from sufficient number of cells. The percentage of adhesion was then calculated by dividing the number of adherent iRBCs to the total number of cells tested. Figure 2(A) shows the percentage of adhesion of each group.

At room temperature (23 °C), 58.9% iRBCs adhered to the CHO-CSA cells. At 37 °C, the percentage of adhesion was 58.4%. There is no significant difference between these two groups. However, after 1 h incubation at 40 °C, the percentage of adhesion increased significantly to 85.6% (p < 0.0001).

We used the dual-pipette step-pressure to quantify the cell-cell adhesion force between the iRBCs and CHO-CSA cells. Figure 2(B) shows the adhesion force measured at 23 °C, 37 °C and after 1 h febrile temperature incubation. At 23 °C, the adhesion force was 148.1 ± 84.2 pN, which slightly decreased to 139.1 ± 85.6 pN at 37 °C (p > 0.05). Strikingly, we observed a significant rise in the adhesion force to 196 ± 105 pN (p < 0.0001) after 1 h incubation at 40 °C.

### Febrile temperature incubation enables a larger contact diameter between the two cells

If the iRBC detachment is considered as a peeling process (process of reducing contact area), the resultant adhesion force is proportional to the contact diameter and the adhesion energy density according to the Young equation. In turn, the contact diameter is proportional to the receptor-ligand density, the bond strength, and the cell membrane property. Furthermore, the adhesion energy density quantifies the unit strength of an adhesive interface, which is proportional to both the bond density and the strength of a single bond^25,26,30,31^. Since 1 h incubation at 40 °C significantly increases the cell-cell adhesion force, we studied the effect of febrile incubation on contact diameter and adhesion energy density.

Figure 1(B) shows the procedure of one iRBC being detached from the CHO cell by the continuous increasing aspiration force. The contact diameter was directly measured from the optical microscopy images with resolution down to 200 nm. Figure 1(C) shows the contact diameter corresponding to different aspirating pressures. With a pressure increase from 40 Pa to 100 Pa, there was only a 20% reduction in the contact diameter. When the aspirating force reached the threshold pressure, the two cells detached completely. The percentage of adhesion (Fig. 2(A) was calculated by dividing the number of adherent iRBCs to the total number of cells tested. Figure 2(B) shows the percentage of adhesion of each group tested.

To quantify the iRBC spreading ability on the CHO-CSA cell, the initial contact diameters were measured and compared among the three temperature groups. Figure 2(C) shows the initial contact diameter measured at 23 °C, 37 °C and after 1 h incubation at 40 °C, respectively. The initial contact diameter that was formed at 23 °C was 1.6 ± 1.2 µm and 1.9 ± 1.24 µm at 37 °C with no significant difference between these two groups (p > 0.05). However, after 1 h incubation at 40 °C, there was a significant increase to 2.49 ± 0.97 µm (p < 0.0001) in contact diameter. We then measured the final contact diameter just before the iRBC was completely separated from the CHO-CSA cell. Figure 2(D) shows the final contact diameter prior to detachment measured at 23 °C, 37 °C and after 1 h incubation at 40 °C were 1.34 ± 0.73 µm, 1.25 ± 0.92 µm and 1.8 ± 0.8 μm, respectively. The latter was significantly larger than the value measured at both 23 °C and 37 °C.

The adhesion energy density is defined as the energy to remove a unit contact area of the cell-cell adhesion^25,27^. It was calculated using the Young equation (Eqn. (2)). At 23 °C, the adhesion energy density measured was 35.85 ± 29.52 μJ/m^2^, while at 37 °C, it was 49.05 ± 36.7 μJ/m^2^. After 1 h incubation at 40 °C, it was 43.88 ± 36.49 μJ/m^2^, which showed an apparent lack of change (p > 0.05) among the three groups, as shown in Figure S1.

### Febrile temperature increases the expression of Phosphatidylserine (PS) on the iRBC surface, but PfEMP1 expression remains unchanged

**Figure 3:**
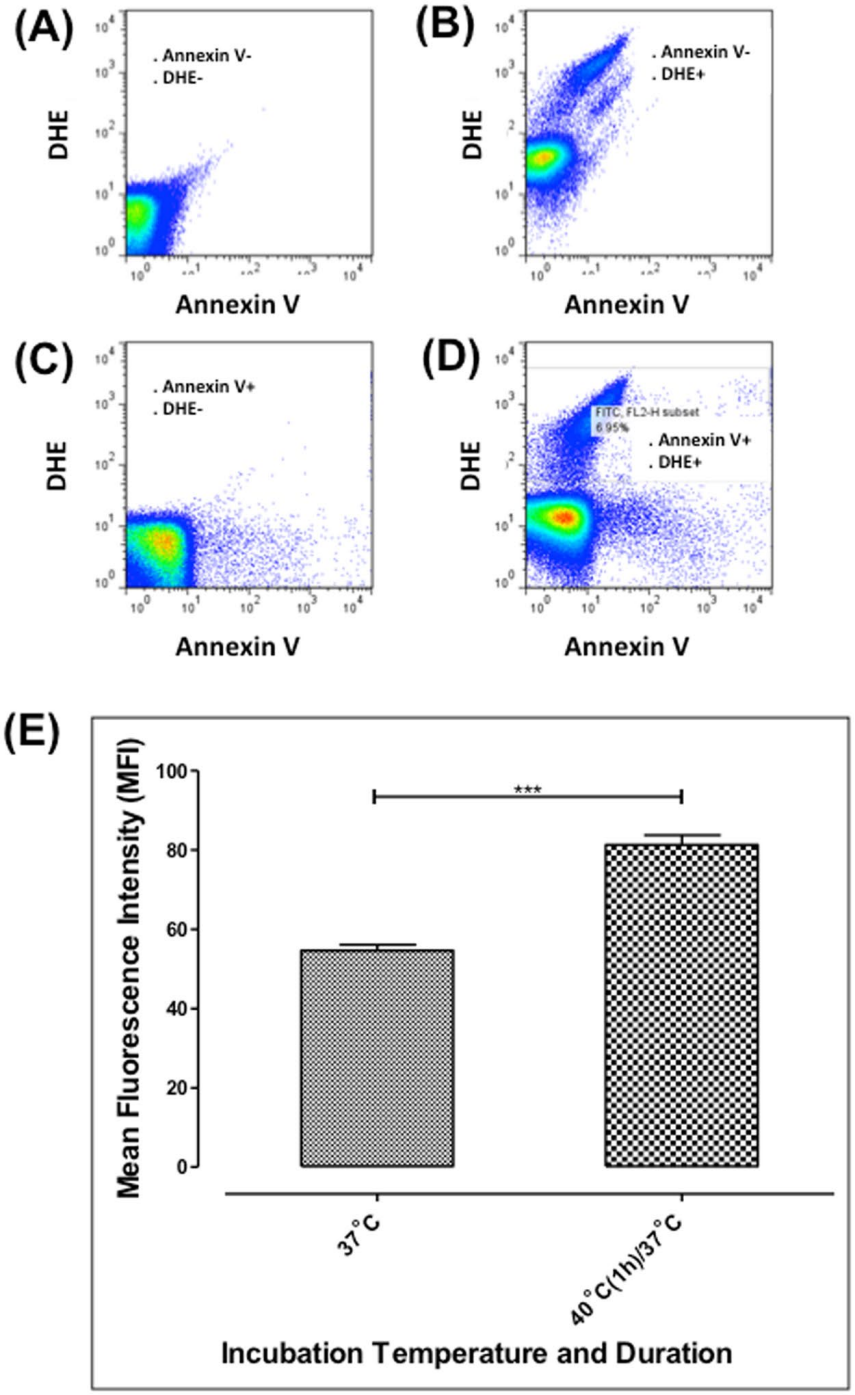
The effect of febrile temperature on the PS expression. (**A**–**D**) Flow cytometric analysis of PS expression. Mean fluorescent intensity (MFI) of Annexin V + DHE + region was quantified. (**E**) Mean fluorescent intensity (MFI) of PS expression of the negative control group and cells incubated at 40 °C for 1 h (n = 3). Mann-Whitney test.

Febrile temperature is known to alter several molecular pathways in iRBCs^21^. Moreover, previous studies have shown that febrile temperature changed the asymmetry of phospholipids on the RBC membrane lipid bilayer and subsequently increased the exposure of PS from the inner-leaflet^16,17^. However, at trophozoite and schizont stage, the expression of *Pf*EMP1 did not change with febrile temperature incubation^21,32,33^. We used flow cytometry to evaluate any changes in PS and *Pf*EMP1 expression on magnet- isolated iRBCs (with ~ 80% purity) incubated at febrile temperature using annexin V and antisera against *Pf*EMP1, respectively (Fig. 3(A–D)). The mean fluorescent intensity (MFI) was measured before and after the fever temperature incubation (Fig. 3(E)). After one-hour incubation at 40 °C, the MFI value of PS increased from 54.67 ± 2.51 to 81.23 ± 5.88. However, one-hour incubation resulted in no significant changes in the MFI of *Pf*EMP1 (37.12 at 37 °C, and 38.22 after 1 h incubation at 40 °C)^33^. A previous study indicates that an extended 24 h-incubation at 40 °C can enhance the PS expression of normal RBCs (nRBCs) significantly^16^. In our experiments (with 2 h incubation), we did not observe any changes of the PS expression on nRBCs upon febrile temperature exposure.

### The increased iRBC adhesion is likely due to the additional negative charges rendered by elevated surface expression of PS

Our results show a significant increase in the adhesion force between iRBCs and CHO-CSA cells upon one-hour incubation at 40 °C. It appeared that the elevated adhesion force arose primarily from the formation of a larger cell-cell contact diameter. However, the adhesion energy density was similar prior to and after incubation at febrile temperature. *Pf*EMP1 and PS are both potential adhesive ligands as reported in previous literature, and the incubation at febrile temperature significantly increased PS expression. Hence, to examine the roles of each adhesive ligand in the increased resultant adhesion force, specific inhibition assay was performed. The adhesion mediated by *Pf*EMP1 was blocked by using soluble CSA, and Annexin V was added to inhibit the adhesion mediated by PS. The adhesion force and percentage were measured after CSA and Annexin V were added.

**Figure 4:**
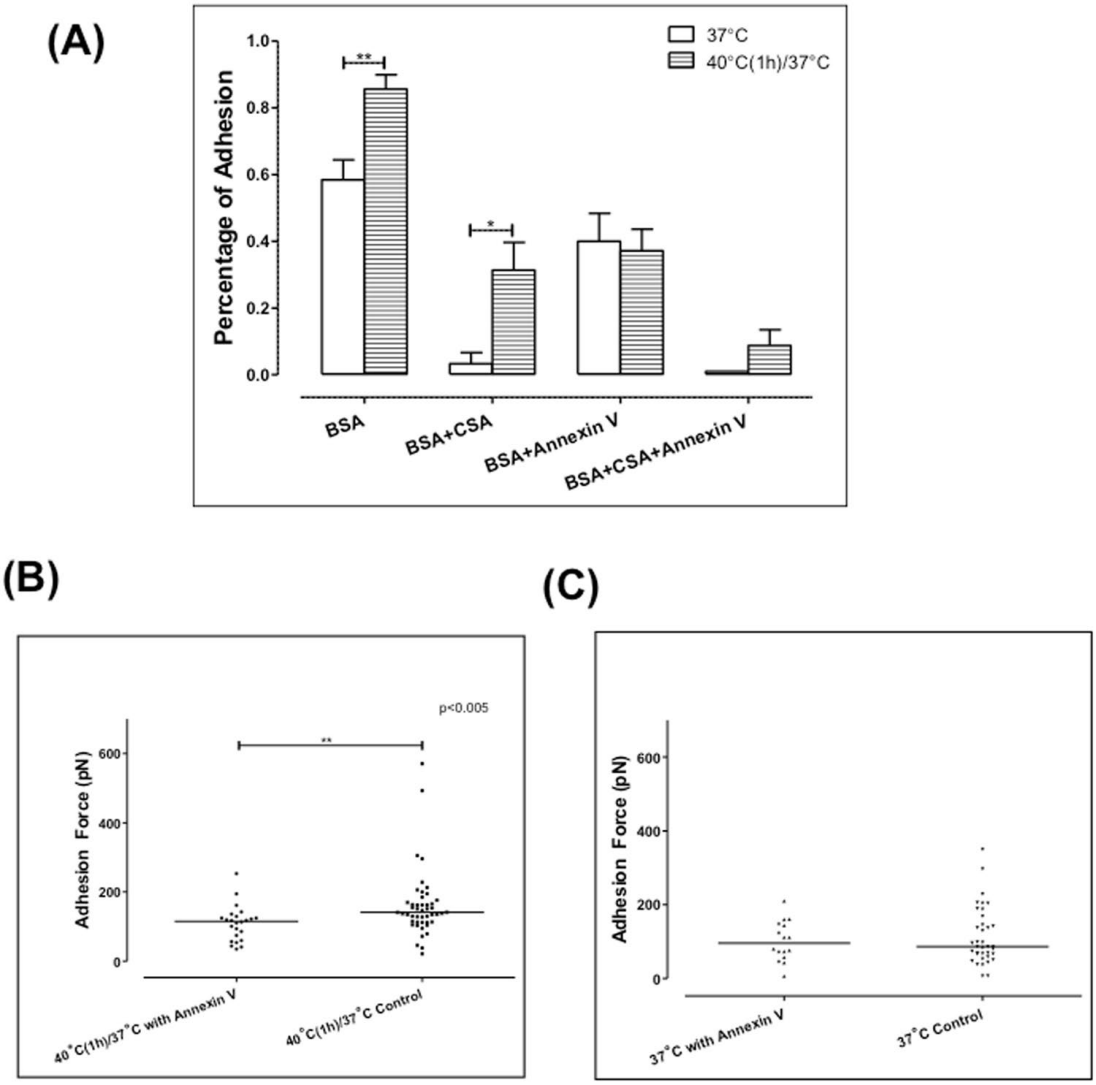
Annexin V significantly inhibited the adhesion after one-hour febrile temperature incubation. (**A**) Percentage of adhesion with CSA and Annexin V (n = 5). (**B**) The adhesion force measured with Annexin V after febrile temperature incubation. (**C**) The adhesion force measured with Annexin V at 37 °C. Each point in (**B** and **C**) represents the adhesion force of one cell pair, and the bar represents the median value of each data set. Mann-Whitney test.

Figure 4(A) shows the percentage of adhesion after the iRBCs being inhibited by CSA and Annexin V. Based on prior literature^34–36^, we used soluble CSA as the adhesion inhibitor in the assay. At 37 °C, soluble CSA reduced the adhesion percentage from 58.4% to 8% while Annexin V reduced the adhesion percentage to 40%. After one-hour incubation at 40 °C, 85.6% iRBCs adhered to CHO cells. With the addition of CSA, the adhesion percentage was reduced to 31.3%, and Annexin V reduced the adhesion percentage to 37.2%. When CSA and Annexin V were combined, adhesion was totally reduced by about 10% in infected RBCs, but remained unchanged in healthy controls.

To examine the adhesion force mediated solely by *Pf*EMP1, Annexin V was added, which specifically blocks any adhesion mediated by PS. The adhesion force was compared with the control group. Figure 4(B,C) show the adhesion force measured with the addition of Annexin V. After one-hour incubation at 40 °C, the adhesion force increased significantly from 113.7 ± 77.43 pN to 159.9 ± 96.33 pN (p < 0.005). However, with the addition of Annexin V, the adhesion force was 102 ± 53.92 pN measured at 37 °C, and it was 108.63 ± 50.45 pN after 1 h incubation. The adhesion force did not change significantly (p > 0.05). We also tried to measure the iRBCs adhesion force mediated by PS only. However, with the addition of soluble CSA blocking CSA-specific bindings, the adhesion force mediated by PS was too weak (below 14 pN) to be measured by the dual-pipette technique.

## Discussion

Cyto-adhesion of iRBCs has important clinical significance during the course of malaria infection, but the impact of recurring fever on iRBCs adherent properties has not been thoroughly studied. Upon parasite maturation, the release of merozoites, metabolic intermediates and other toxic molecules stimulate the production of pyrogenic inflammatory mediators and cytokines. Subsequently, these cytokines send signals to the brain to elevate the core body temperature^21,37–40^. Hence, in a synchronized iRBC population, the fever that starts with iRBC rupture only affects the ring stage parasites. However, in acute *falciparum* malaria infections, asynchronous or even bimodal distributed parasites exist in patients^41–45^. Thus, elevated body temperature caused by the fever could also affect adhering trophozoites and fully developed schizonts. A short period of exposure to febrile temperature significantly induces more iRBCs to adhere to endothelial receptors, including the normally non-adhesive ring stage iRBCs^32,33^. A brief febrile incubation accelerates the expression of P*f*EMP1, and the appearance of P*f*EMP1 at ring stage enhances fever-induced ring stage adhesion. However, at trophozoite stage, the P*f*EMP1 expression was not altered^21,32^. Thus, the possibilities of other proteins involved in fever-induced adhesion are not ruled out.

Here we used the dual-pipette step-pressure technique to quantify the stable cell-cell adhesion of *P. falciparum* iRBCs and CHO cells expressing CSA (VAR2CSA). CSA is one of the major ligands for placental adhesion of human plasmodia, and it could be the basis for a vaccine against pregnancy malaria^46^. Thus, we used the parasite line that has been selected for CSA binding, and the CHO cells as surrogates to the human host cells. Overall, our results demonstrated a two-fold increase in the adhesion force as well as a larger cell-cell contact area formation between trophozoite stage iRBCs and target CHO-CSA cells at elevated temperature. Correspondingly, the adhesion percentage increased as well. Moreover, we identified that the increased adhesion was derived from an increased exposure of PS on the iRBC surface under thermally stressed condition. The expression level of *Pf*EMP1 remained unchanged, as determined by FACS. PS is a negatively charged phospholipid, and any altered exposure of PS changes the cell membrane charge balance rendering a more adhesive cell surface^14,47^. Specifically, PS can bind to several host receptors including TSP and CD36, and it is one of the non-specific adhesive ligands involved in malarial cytoadherence as well as sickle cell disease^9,11,14,15^. Recent studies suggested that at febrile temperature, the lipid bilayer loses its phospholipids asymmetry and as a result, the PS components flip to the membrane outer layer^16,17^. Thus, during febrile temperature, increased surface expression of PS significantly enhanced the cell-cell adhesion strength and allowed more iRBCs adhering to endothelial cell receptors. Our quantitative measurements showed that a 50% increase in the adhesion force could be brought by the increased PS exposure at febrile temperature for iRBCs. Point contacts were observed between healthy RBCs to CHO cells after febrile temperature incubation, but the adhesion force was below the threshold of the dual pipette step-pressure technique (below 14 pN). The point contacts were inhibited by the addition of Annexin V (Fig. S2). A significant PS expression in normal RBCs is expected only upon prolonged febrile incubation^16^.

The cell-cell adhesion of receptor-ligand interactions is dominated by several factors. Here we used the “fracture energy density” approach^25,26^ to describe the adhesion process. In this approach, the Young equation (Eqn. (2)) indicates that the resultant cell-cell adhesion force is proportional to the contact diameter and the adhesion energy density. Being exposed to febrile temperature greatly enhanced the PS expression; therefore, larger contacts were formed between two infected cells. The adhesion energy density is defined as the energy to separate a unit contact^25–27,48^, and it is proportional to both the adhesive ligand density and the strength to break a single receptor-ligand pair^25,31^. While the febrile temperature incubation enhanced the non-specific adhesive ligand (PS) expression, it did not change the adhesion energy density significantly. Similar to what we observed in the present study (Fig. 4(A)), increased binding frequency between iRBCs and CHO-CSA cells was found by atomic force microscopy (AFM) at febrile temperature in our recent study^33^. Considering the small (~20%) drop in specific CSA-P*f*EMP1 single receptor-ligand pair binding force^33^, it appears that increased non-specific ligand (PS) expression balanced the weakened single and multiple CSA-P*f*EMP1 bindings with no changes in P*f*EMP1 expression after febrile temperature incubation. Thus, the overall adhesion energy density did not change significantly. Nevertheless, much stronger overall adhesion force between the two cells was observed with the increased PS expression. The dual-pipette cell-cell adhesion assay is ideal for capturing the overall adhesion rupture force between two cells, which can be used to give a more complete picture together with the specific single receptor-ligand rupture force measurements.

Recent *in vitro* studies have suggested that the fever episodes contribute to increased parasite clearance and thus it is protective to the human host. Parasites exposed to high fever proceeds to an apoptosis-like progressive cell death pathway^21^. With their surface exposed to PS, these iRBCs are ready to be recognized and cleared by phagocytes in spleen^49–52^. Hence, malaria-associated fever is considered beneficial to the patients suffering from the disease. Some but not all studies have shown that antipyretic drugs, with their ability of alleviating fever, extend the parasites clearance time^53,54^. Although longer exposure to febrile temperature (>2 h) leads to parasites death, we demonstrate that a short exposure (<1 h) in fact significantly enhanced the iRBCs adherence via PS. Increased surface expression of PS could possibly contribute to the parasite clearance through apoptosis-like pathway. In addition, the stronger adhesion force can facilitate parasites sequestration and help them escape mechanical barriers associated with splenic clearance. As a result, it supports long-term parasite survival and at the same time keeps low levels of infection. Although these *in vitro* studies provide direct evidence to the effect of febrile temperature on cytoadherence by malaria-iRBCs, adhesion measurements were performed in static environment. Further experiments using dynamic modes of measurements and the use of *in vivo* model systems will strengthen the current understanding of the relationship between febrile temperature and malaria cytoadhesion.

## Conclusion

Here, we report a link between febrile temperature, PS expression and the elevated adhesive properties of malaria-iRBCs. Our results demonstrate that a short exposure to febrile temperature significantly increases both the adhesion force and adhesion percentage between iRBCs and CSA-CHO cells, which might be related to clinical manifestations relevant to malaria-associated fever.

## Electronic supplementary material

Supplemental Figures
