# Supplementary material for: Febrile Temperature Elevates the Expression of Phosphatidylserine on Plasmodium falciparum (FCR3CSA) Infected Red Blood Cell Surface Leading to Increased Cytoadhesion
Source: Sci Rep. 2018 Oct 9;8:15022. doi: 10.1038/s41598-018-33358-2 (PMC6177484; doi:10.1038/s41598-018-33358-2)
Supplement: Supplementary file 1 — Supplemental Figures [file 41598_2018_33358_MOESM1_ESM.pdf]

Title: Febrile Temperature Elevates the Expression of Phosphatidylserine on Plasmodium falciparum (FCR3CSA) Infected Red Blood Cell Surface Leading to Increased Cytoadhesion

Rou Zhang, Rajesh Chandramohanadas*, Chwee Teck Lim* and Ming Dao*

[1] Singapore-MIT Alliance, National University of Singapore, Singapore 117576

[2] Infectious Disease IRG, Singapore-MIT Alliance for Research and Technology (SMART) Centre, Singapore 117543

[3] Department of Bioengineering, National University of Singapore, Singapore 117576

[4] Engineering Product Development Pillar, Singapore University of Technology & Design, 20 Dover Drive, 138682 Singapore

[5] Department of Materials Science and Engineering, Massachusetts Institute of Technology, Cambridge, MA 02139

[*] Corresponding authors: Rajesh Chandramohanadas (rajesh@sutd.edu.sg), Chwee-Teck Lim (ctlim@nus.edu.sg) and Ming Dao (mingdao@mit.edu)

Supplemental Figure. S1

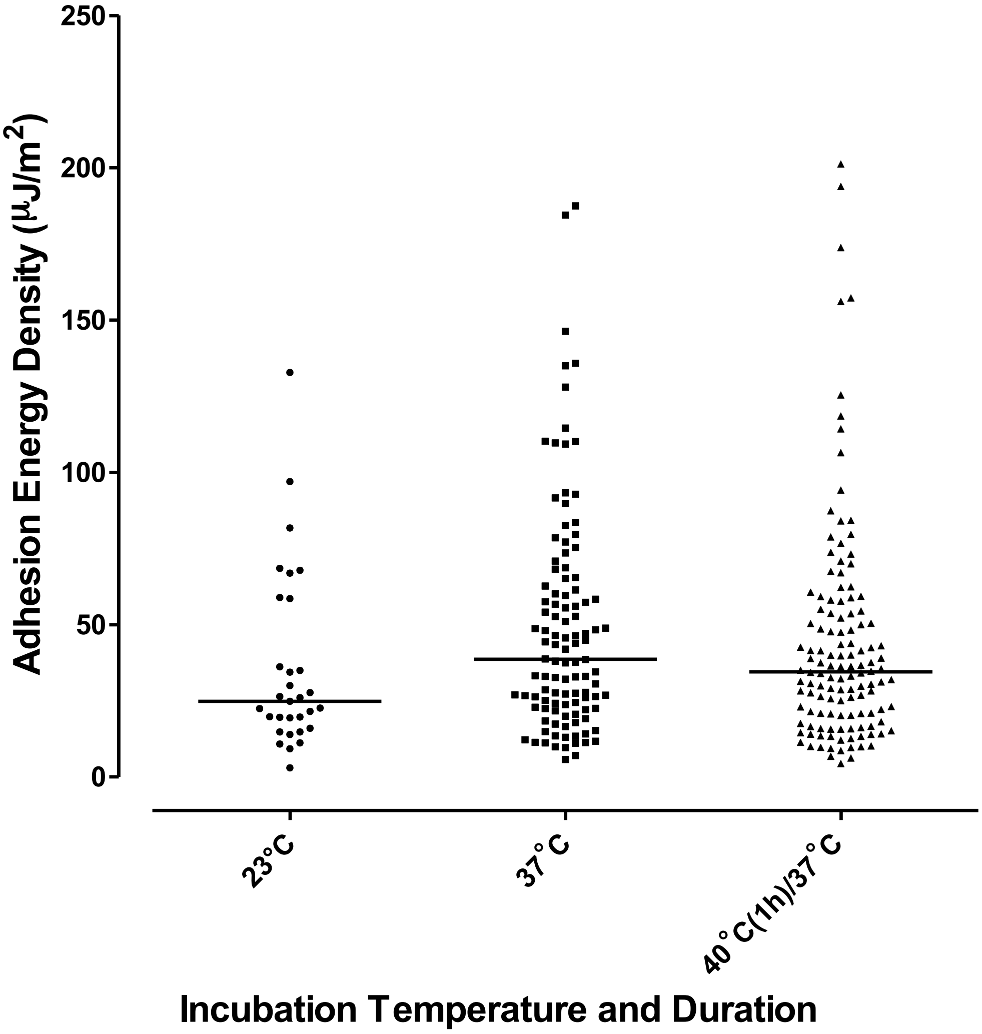

Figure S1. The effect of febrile temperature on the cell-cell adhesion energy density. Measurements were done at 23°C, 37°C, and after 1 h incubation at 40°C. Each point represents the adhesion energy density of one cell pair. Graphs show the median, standard deviation, minimum and maximum values of the adhesion energy density. Krauskal-Wallis Test and Dunn's post hoc test.

Supplemental Figure. S2

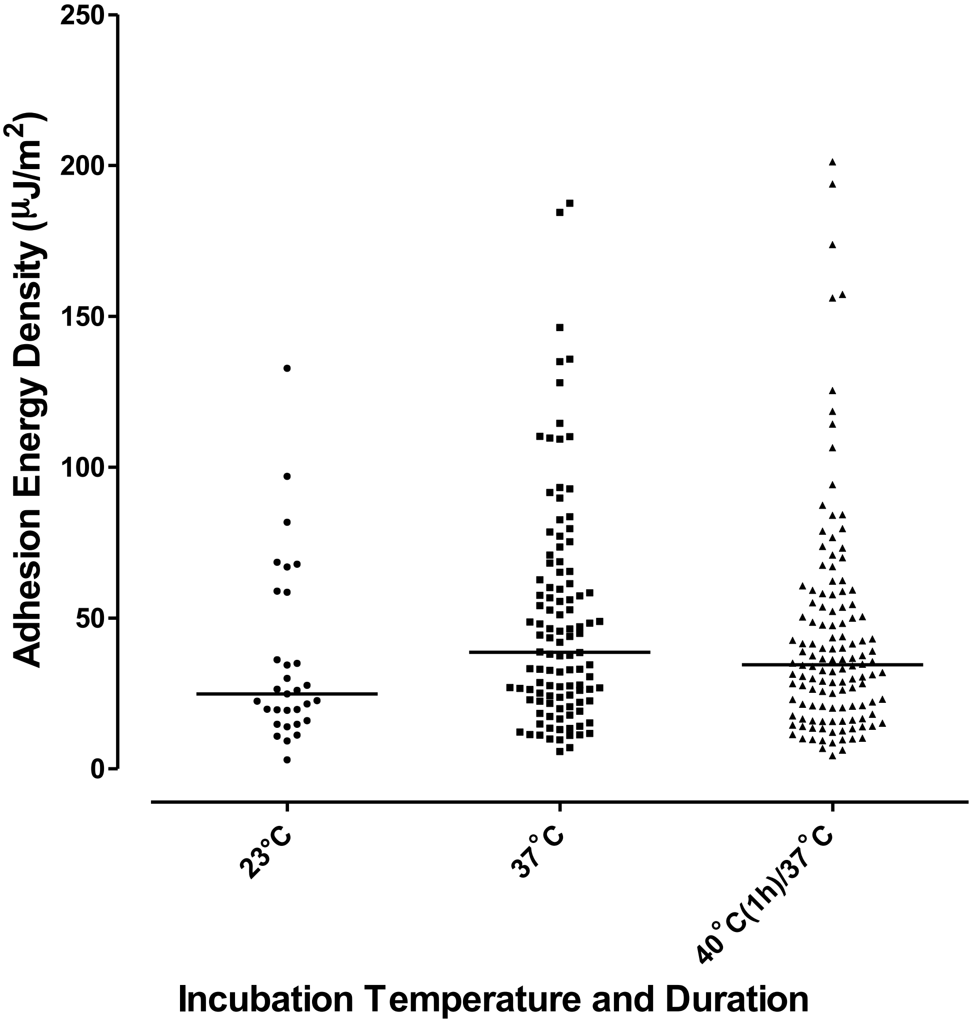

Figure S2. Febrile temperature incubation greatly enhanced point contact formation between nRBCs and CHO cells. The point contacts were inhibited by the addition of Annexin V.
